# All STEM fields are not created equal: People and things interests explain gender disparities across STEM fields

**DOI:** 10.3389/fpsyg.2015.00189

**Published:** 2015-02-25

**Authors:** Rong Su, James Rounds

**Affiliations:** ^1^Department of Psychological Sciences, Purdue UniversityWest Lafayette, IN, USA; ^2^Department of Educational Psychology and Psychology, University of Illinois at Urbana-ChampaignChampaign, IL, USA

**Keywords:** interests, gender differences, people-orientation, things-orientation, gender disparities in STEM fields

## Abstract

The degree of women's underrepresentation varies by STEM fields. Women are now overrepresented in social sciences, yet only constitute a fraction of the engineering workforce. In the current study, we investigated the gender differences in interests as an explanation for the differential distribution of women across sub-disciplines of STEM as well as the overall underrepresentation of women in STEM fields. Specifically, we meta-analytically reviewed norm data on basic interests from 52 samples in 33 interest inventories published between 1964 and 2007, with a total of 209,810 male and 223,268 female respondents. We found gender differences in interests to vary largely by STEM field, with the largest gender differences in interests favoring men observed in engineering disciplines (*d* = 0.83–1.21), and in contrast, gender differences in interests favoring women in social sciences and medical services (*d* = −0.33 and −0.40, respectively). Importantly, the gender composition (percentages of women) in STEM fields reflects these gender differences in interests. The patterns of gender differences in interests and the actual gender composition in STEM fields were explained by the people-orientation and things-orientation of work environments, and were not associated with the level of quantitative ability required. These findings suggest potential interventions targeting interests in STEM education to facilitate individuals' ability and career development and strategies to reform work environments to better attract and retain women in STEM occupations.

## Introduction

Despite major advancement of women's participation and status in the workforce over the past decades, women overall remain the minority in science, technology, engineering, and mathematics (STEM) disciplines. The underrepresentation of women in STEM fields keeps our society from fully utilizing human capital and is of great concern to researchers, educators, and the general public. However, past research on this topic typically treated all STEM fields as a whole and ignored the differences among sub-disciplines of STEM. It is important to note that all STEM fields are not identical. Sub-disciplines of STEM vary in their culture and climate, training and preparation required, and the type of work activities involved. The percentages of women across subfields of STEM also vary vastly. For example, women have made immense progress in biomedical and social sciences, now earning over 50% of bachelor's and master's degrees, whereas the percentage of women obtaining any level of engineering degree lingers below 20% (National Science Foundation, 2013). To build a more balanced and competitive workforce, we need to gain a better understanding about the psychological and socio-cultural factors that contribute to the differential participation of women across STEM sub-disciplines. Investigating why women are scarce in some STEM fields but not in others may offer us insight into how to increase women's overall representation in STEM.

The current study focuses on the differential interests of men and women that may drive career choices within STEM fields just as they influence the selection between STEM and other careers^1^. Interests have been consistently shown as a critical predictor for career choice and career attainment. Existing studies have suggested that the differential interests of men and women are one of the most important psychological mechanisms that underlie gendered career choices and gender disparities in the STEM fields (e.g., Lubinski and Benbow, 1992; Ceci et al., 2009; Su et al., 2009). For example, Su et al. (2009) examined gender differences in vocational interests and two work-task dimensions (namely, *Things–People* and *Data–Ideas*; Prediger, 1982) and found substantial gender differences in the *Things–People* dimension (*d* = 0.93), with men preferring working with things and women preferring working with people. The effect size of this gender difference in interests was close to one standard deviation, and among the largest reported in the literature of individual differences (Lubinski, 2000). Interests in people-oriented careers may explain women's underrepresentation in some STEM fields, which are typically things-oriented.

Despite these findings suggesting the role of interests in gendered career choices, several gaps exist in this research. First, no study has looked within STEM fields and investigated men and women's interests in each sub-discipline of STEM. Second, although many studies reported statistics on the percentages of women in STEM occupations, no research has compared the trend in labor statistics with gender differences in interests to examine whether actual percentages of women in each STEM sub-discipline match or mismatch their interests. This information is critical, as it will help identify areas where interventions could be fruitful for increasing the participation of women. Third, past research typically studied the determinants of STEM career choices using individuals as the unit of analysis and rarely incorporated indicators of occupational characteristics to study their effects on men and women's interests at the occupational level. Understanding the interaction between individuals' interests and the characteristics of STEM occupations is essential for explaining why women remain severely underrepresented in some STEM fields and yet are growing in numbers in other STEM fields that are equally demanding intellectually and temporally.

In this article, we seek to advance the literature in the following ways. First, we highlight the differential interests of men and women within STEM fields and offer it as one explanation for the uneven distribution of women across the STEM disciplines. We extended Su et al. (2009) meta-analysis and examined gender differences in basic interests (i.e., specific and homogeneous interests in activities and objects with shared properties, such as *Mathematics* or *Biological Science*). Specifically, we examined men's and women's basic interests in the full range of STEM fields, from Engineering in which the number of women are the sparsest, to Social Sciences in which women are over-represented. Further, we investigated the extent to which gender differences in basic interests contributed to the gender composition (percentages of men and women) in corresponding occupational fields, and the degree to which these gender differences in basic interests mediated the effects of occupational characteristics, such as people- and things-orientation and job requirement in quantitative ability, using a person–environment (P–E) fit approach.

## The other side of the coin: the role of interests in STEM careers

Person–environment (P–E) fit theories (e.g., Holland, 1959, 1997; Pervin, 1968; Dawis and Lofquist, 1984; Schneider, 1987) maintain that individuals and environments can be described using a commensurate set of characteristics. For example, an individual can be described in terms of his/her interests in social or people-related activities, and an environment can be described in terms of its likelihood to fulfill such interests. An environment may be conceptualized at a variety of different levels, such as an academic major, an occupational field, organizational culture and climate, or the relationship with supervisor and work team (Su et al., 2014). Further, the degree of compatibility between individual and environmental characteristics is associated with career choice, satisfaction, and performance. Individuals seek out and thrive in environments that provide a good fit with their traits and motives; they are likely to stay in environments that are compatible, and will leave those environments that are incompatible. As such, people's interests in work environments channel their career decision-making and career advancement.

It has been consistently shown that, compared to men, women have stronger preference for work environments that provide more opportunities and activities to work with people. Such preference has been explained under different theoretical frameworks, such as people-orientation (e.g., Thorndike, 1911; Woodcock et al., 2013), social interests (e.g., Su et al., 2009; Robertson et al., 2010), subjective task values (e.g., Meece et al., 1982; Eccles, 2007), and communal goals (e.g., Diekman et al., 2010; McCarty et al., 2014). Regardless of the theoretical framework used, research in this area has shown that differential preferences of men and women are associated with the gender disparities in STEM fields. For example, in a series of 15 studies, Woodcock et al. (2013) examined the people-orientation and things-orientation of 7450 participants and found that females consistently scored higher than males in people-orientation (mean *d* = 0.49, range from 0.11 to 0.86), whereas males consistently scored higher than females in things-orientation (mean *d* = 0.99, range from 0.58 to 1.33). Moreover, Woodcock et al. (2013) showed that people- and things-orientations predicted the choice of a STEM major in college, with things-orientation positively associated with STEM major choice and people-orientation moderating this relationship (that is, a particularly strong relationship between things-orientation and STEM major choice when people-orientation is low). The effects of people- and things-orientations on STEM major choice fully accounted for the effect of sex.

Similarly, Su et al. (2009) conducted a meta-analysis that quantitatively synthesized data from 47 interest inventories with 503,188 respondents, and reported substantial gender differences in interests. Specifically, males on average scored higher on the Realistic scale that measured interests in working with things and gadgets or working outdoors (*d* = 0.84); in contrast, females on average scored higher on the Social scale that measured interests in helping people (*d* = −0.68). Su et al. (2009) argued that gender disparities in STEM fields occurred for two reasons: first, from an inter-individual perspective, men outnumber women in the upper tail of the Realistic interest distribution, which predicts entry into things-oriented careers including STEM fields; second, from an intra-individual perspective, given the same level of Realistic interests, women are more likely than men to have a competing level of Social interests, which orient them toward people-oriented careers, or, within STEM fields, those sub-disciplines that are more likely to fulfill their interests in helping people, such as medical science and services.

Eccles and her colleagues (Meece et al., 1982; Eccles, 1994, 2007, 2009; Jacobs et al., 2005) argued that the perceived task values of various occupational options (e.g., “Can I directly relate to people and help people in this occupation?”) is one of the most important mechanisms underlying educational and occupational choices, including the decision to enter STEM fields and the choice within various STEM sub-disciplines. Because females are socialized to possess higher social values in interacting and helping people, they are more likely to be drawn to occupational fields with work tasks that are perceived to fulfill these values, such as teaching, nursing, or medical science, rather than fields that are perceived to be low in these values, such as physical science and engineering.

Lastly, through two experimental studies, McCarty et al. (2014) demonstrated that participants who highly valued communal goals, regardless of gender, had aversive and avoidant reactions to work environment that is low in communion. Specifically, Diekman et al. (2010, 2011) showed that the endorsement of communal goals significantly impeded intention to pursue STEM careers, even when controlling for past experience and self-efficacy in science and mathematics. Consistent with the literature, women on average scored higher on communion than men, suggesting that women were less likely to favor work environments that are perceived less compatible with communal goals, including some STEM fields.

Based on the above evidence, we argue that men and women's differential interests for work environments provide the other side of the coin—an equally, if not more, important psychological mechanism for understanding the gender disparities in STEM fields—in addition to cognition and learning pertaining to math preparation and achievement. We propose that the interests that underlie women's overall underrepresentation in STEM fields also underlie the differential distribution of women across STEM disciplines and explain why women tend to choose some STEM disciplines over others.

## The conceptualization, function, and measurement of interests

Interests are defined as “trait-like preferences for activities, contexts in which activities occur, or outcomes associated with preferred activities” that orient individuals toward certain environments and motivate goal-oriented behaviors within environments (Rounds and Su, 2014). Such intrinsic preferences construe an essential part of individuals' identity and serve as an impetus for individuals to navigate through and function effectively in their environments (Hogan and Blake, 1999; Su et al., 2009).

Based on P–E fit theories (e.g., Holland, 1997), interests directly influence educational and career choices as people gravitate toward academic or work environments that are congruent with their interests. It has been reliably shown that interests predict academic major and occupational membership (e.g., Strong, 1943; Campbell, 1971; Kuder, 1977; Savickas and Spokane, 1999). In addition, interests also impact career trajectory and attainment through its indirect effects on learning and knowledge acquisition, which prepares as well as constrains one's pursuit in certain educational and occupational fields. Interests in an activity act as a source of intrinsic motivation that drives individuals to learn more about it. An accruing volume of research has linked interest with persisted learning, deeper engagement, and better knowledge acquisition (e.g., Hidi, 2001; Silvia, 2006) and has shown the increasing coupling of interests and domain-specific knowledge/ability over time (Ackerman, 1996; Denissen et al., 2007). Thus, an individual with strong interests in mathematics, for example, is more likely than his/her uninterested peers to aspire to education and a career in mathematics; in the meanwhile, this individual is more likely to engage in activities to learn math that leads to increased math knowledge and ability, which, in turn, prepares him/her for entry into a math major or a math-related career as well as persistence and attainment in that field.

This dynamic relationship between interests and knowledge/ability development is critically important for understanding the significance of interests for educational and career attainment in STEM. Interests do not only serve as a self-selection mechanism for a few binary choices in life such as choosing a college major or entering an occupation; rather, interests contribute to individuals' preparedness for STEM fields by promoting learning in these fields and provide the foundation for individuals' educational and career development throughout the lifespan.

Interests can be conceptualized and measured at different levels of specificity. The most commonly studied interest typology is Holland's (1959, 1997) RIASEC model (abbreviation for *Realistic, Investigative, Artistic, Social, Enterprising*, and *Conventional*), which is used to categorize both individual interests and corresponding characteristics in work environments. The RIASEC model captures the broadest level of interests and work environments. Each of the six broad categories encompasses a heterogeneous group of occupations and activities that share a common “theme.” Therefore, the RIASEC types are sometimes also referred to as the general occupational themes. For example, the *Realistic* (R) theme captures interests in working with things and gadgets, working with hands, or working outdoors. Typical occupations and work activities represented in the *Realistic* theme include carpenters, automotive engineers, farming, or putting out forest fires. The *Social* (S) theme captures interests in working with people and helping people. Typical occupations and work activities included in the *Social* theme are teachers, social workers, volunteering at a charity, or helping people solve their emotional problems. Realistic and social interests are closely associated with the constructs of things- and people-orientations (Woodcock et al., 2013). The *Investigative* (I) theme, as its name suggests, captures interests in science and research. It is the best indicator for the interests in pursuing education or careers in STEM fields. However, STEM is a broad term with heterogeneous sub-disciplines. Many disciplines in natural sciences, such as physical science, astronomy, and chemistry, also involve a heavy *Realistic* component; the most quintessential is the field of engineering, with a strong focus on working with things, in addition to its emphasis on research and investigation; in comparison, other disciplines in health and human sciences, such as psychology, medicine, or nutrition science, also involve a *Social* component. Therefore, although most STEM fields fall within the *Investigative* theme, they may be arranged on a continuum from the most things-oriented to the least things-oriented field, and from the most people-oriented to the least people-oriented. The broad occupational themes are not sufficient to capture the nuances among various sectors in the world of work and the heterogeneous interests represented in these environments. More specific measures of interests are needed.

Basic interest scales characterize shared properties of homogeneous sets of work activities and environments (Liao et al., 2008). For example, instead of broad *Social* interests, a basic interest scale may measure interest in *Teaching, Counseling*, or *Professional Advising* activities; similarly, instead of broad *Investigative* interests, a basic interest scale may measure interest in *Mathematics, Physical Science*, or *Medical Science* activities. What is unique about basic interest scales is that the interest measured by a basic interest scale is often implied in the object of interest. A *Medical Science* basic interest scale may include items like “work in a lab,” “study blood samples,” and “develop a new medicine to cure a disease.” Taken together, responses to these items reflect an individual's level of interest in the field of medical science. In other words, the specificity of basic interests corresponds precisely with the targeted environments. As a result, basic interests provide an excellent measure of individuals' preferences for specific work environments; gender differences in basic interest scales represent differential preferences of men and women for these work environments, such as sub-disciplines of STEM.

## Overview and hypotheses of the current study

The purposes of the current study were three-fold: first, we examined gender differences in basic interests by STEM field, including physical sciences, biological science, medical science, medical services, social sciences, mathematics, applied mathematics, computer science, engineering, and mechanics and electronics. Because the definition of STEM disciplines varies by organization and a unified list is not available, we adopted the definition from two federal agencies: (1) *STEM-Designated Degree Programs List* from the U.S. Immigration and Customs Enforcement (2012), and (2) *STEM Workforce Sectors* from the U.S. Department of Labor (2007). We expected gender differences in basic interests to vary largely across these different STEM disciplines. Second, we demonstrated that the gender composition (percentages of men and women) in these STEM fields closely mirrored the pattern of gender differences in basic interests. Third, we sought to understand the occupational characteristics that were associated with the gender differences in basic interests.

To answer these research questions, we meta-analytically reviewed technical manuals of interest inventories that included a relevant basic interest scale. Because traditional meta-analysis is subject to sampling errors from individual studies reviewed, we selected norm groups from technical manuals to be our data source as they are typically large and well sampled (cf. Hedges and Nowell, 1995). Data from these technical manuals provide relatively accurate estimation of the differential interests of men and women for each sub-discipline of STEM. In addition, we obtained occupational characteristics from the Occupational Information Network (O^*^NET; National Center for O^*^NET Development, 2014) on the things-orientation, people-orientation, and level (i.e., amount) of quantitative ability required for each sub-discipline of STEM.

Based on P–E fit theories and existing studies showing that women had higher people interests and lower things interests compared to men, we expected to find greater gender differences in basic interests favoring men in STEM fields that are high in things-orientation and low in people-orientation, such as engineering; we expected to find smaller gender differences in basic interests favoring men or gender differences in the opposite direction in STEM fields that are low in things-orientation and high in people-orientation, such as medical and social sciences. Consistent with the continuum of STEM sub-disciplines ordered by their things- and people-orientations, we expected the size of gender differences in basic interests in these fields to form a continuum as well. Given previous research reporting that people-orientation and things-orientation are two separate dimensions instead of opposite ends of one bipolar dimension (e.g., Graziano et al., 2011; Tay et al., 2011), we propose the following two hypotheses:

*Hypothesis 1a*: The gender difference in interests in a STEM field (favoring men) is positively associated with the level of things-orientation of that field.
*Hypothesis 1b*: The gender difference in interests in a STEM field (favoring men) is negatively associated with the level of people-orientation of that field.

With research evidence showing that gender differences in math ability and achievement are negligible (e.g., Hyde et al., 1990; Hyde and Linn, 2006), we reason that quantitative ability is not a factor that affects men and women's differential career preferences. Thus, we expected the gender difference in interests in a STEM field to be unrelated to the level of quantitative ability required for that field once the things- and people-orientations of the field are accounted for. In other words, women's lower interest in some STEM fields is not the result of their avoidance of work environments that require higher levels of quantitative ability, but rather, the result of their aversion to work environments that are high in things-orientation and low in people-orientation.

*Hypothesis 2*. Controlling for the things- and people-orientations of a STEM field, the gender difference in interests in that field is unrelated to the level of quantitative ability required.

More importantly, given the strong relationship found between interests and career choices, we expected the gender composition of various STEM fields to reflect observed gender differences in interests. Further, we expected the gender difference in interests in a STEM field to fully mediate the effects of occupational characteristics (things- and people-orientations) on the gender composition of that field. Similar to Hypothesis 2, we expected the gender composition of a STEM field to be unrelated to the level of quantitative ability required for that field once the things- and people-orientations of the field are accounted for.

*Hypothesis 3*. The percentage of women in a STEM field is negatively associated with the gender difference in interests in that field (favoring men).
*Hypothesis 4a*. The percentage of women in a STEM field is positively associated with the people-orientation of that field; this relationship is full mediated through gender differences in interests.
*Hypothesis 4b*. The percentage of women in a STEM field is negatively associated with the things-orientation of that field; this relationship is full mediated through gender differences in interests.
*Hypothesis 5*. Controlling for the things- and people-orientations of a STEM field, the percentage of women in that field is unrelated to the level of quantitative ability required.

Besides the above hypotheses, we examined several additional moderators for the gender differences in basic interests in STEM fields, including (1) job complexity, (2) the age group of a sample, (3) the year of data collection, and (4) the degree to which an interest inventory was developed to be gender-balanced (i.e., using item development strategies to remove items that displayed large gender differences and to increase the overlap between male and female interest score distributions). More details are provided for these moderators in the Methods section.

## Methods

### Meta-analytic database

Database for the current study was composed of norm samples from vocational interest inventory technical manuals, published from 1964 till the current date. Procedures to identify and select the interest inventories were described in detail in Su et al. (2009). Because we were interested in the gender differences in basic interests in this study, the following criteria were applied to select interest inventories to form the current meta-analytic database: first, the interest inventory had one or more scales that measured *basic interests* in any fields related to physical sciences, biological science, medical science, social sciences, mathematics, computer science, and engineering. We included scales that measured interests in professional-level activities in these fields (i.e., activities performed by scientists, engineers, and mathematicians), as well as scales that measured interests in technical-level activities (i.e., activities performed by science technicians, engineering technicians, workers in applied mathematics, mechanics and electronics, and those in medical services). By including interest scales at both levels in our database, we were able to examine whether job complexity had an effect on the gender differences in basic interests and on the gender composition of various STEM fields. Second, the inventories used the same form for male and female respondents and reported means and standard deviations for both males and females in the technical manuals, allowing effect sizes of gender differences to be calculated. Third, because it was possible for an interest inventory to have multiple editions, we included data from a new edition only when it used an entirely new sample. Application of these inclusion criteria resulted in 52 samples from 33 inventories, with a total of 209,810 men and 223,268 women. The mean ages of the samples ranged from 12.50 to 42.55 years. The samples were surveyed between 1963 and 2007.

### Classification of basic interest scales by STEM field

To identify relevant basic interest scales for each sub-discipline of STEM, we perused every interest inventory and classified each basic interest scale into corresponding STEM field based on (1) the items on the scale and (2) the correlates of the scale score. Most basic interest scales measured interests as suggested by their titles, such as the *Social Science* scale in the Jackson Vocational Interest Survey (JVIS; Jackson, 2000). A few exceptions were classified differently than their title would suggest. For example, the *Engineering and Physical Sciences* scale in the Ohio Vocational Interest Survey II (OVIS-II; Winefordner, 1983) was classified as a scale measuring interests in engineering, rather than physical sciences, because the majority of its items were occupational titles in engineering, such as “Electronics Engineer” and “Nuclear Engineer.” Similarly, the *Mathematics and Science* scale in the Career Interest Inventory (CII; Psychological Corporation, 1991) had mostly engineering and computer science related items and was classified as a scale measuring engineering interests. The *Science* scale in the Vocational Interest Inventory-Revised (VII-R; Lunneborg, 1993) primarily measured interests in medical science, and the *Mechanical* scale in the Guilford-Zimmerman Interest Inventory (GZII; Guilford and Zimmerman, 1989) had items that measured interests in the professional-level of engineering activities, rather than the technical-level of mechanical activities. These scales were classified accordingly.

Further, some basic interest scales measured interests broader than one STEM field. For example, several scales, including the *Science* scale in the Career Assessment Inventory-Vocational edition (CAI-V; Johansson, 1984), measured interests in physical sciences and biological science. A separate category, *Natural Sciences*, was hence created to classify these scales, rather than forcing them into either the *Physical Sciences* category or the *Biological Science* category. Finally, scales that were designed to measure basic interests but rather measured the full range of interests in all disciplines of sciences and research, such as the *Research* scale in the Strong Interest Inventory (SII; Donnay et al., 2005), were excluded from the current study.

As a result, the basic interest scales from all the interest inventories were classified into 13 fields. Eight of these fields were at the professional-level, including *Physical Sciences, Natural Sciences, Biological Science, Medical Science, Social Sciences, Mathematics, Computer Science*, and *Engineering*; the other five fields were at the technical-level, including *Science Technicians, Engineering Technicians, Applied Mathematics, Mechanics and Electronics*, and *Medical Services*. Table 1 lists all the basic interest scales classified under each STEM field by sample.

**Table 1:** **Overview of the meta-analysis database: basic interest scale, moderator variables, and effect size by STEM field and sample**.

**Inventory**	**References**	**Scale**	**Moderator**				**Sample size**			**Effect size**
			**Complexity**	**Gender-balanced**	**Sample year**	**Age group**	***N***	***MN***	***FN***	***d***
**PHYSICAL SCIENCES**										
SII	Donnay et al., 2005	Science	2	1	2002	5	2250	1125	1125	0.42
SII	Harmon et al., 1994	Science	2	1	1994	5	18,951	9484	9467	0.24
SII	Hansen and Campbell, 1985	Science	2	1	1985	5	600	300	300	0.28
SII	Campbell, 1974	Science	2	1	1974	5	600	300	300	0.32
CAI-E	Johansson, 2003	Scientific Research and Development	2	2	1986	5	900	450	450	0.23
KGIS-E	Kuder, 1964	Scientific	2	1	1963	1	4109	2080	2029	0.91
KGIS-E	Kuder, 1964	Scientific	2	1	1963	2	5704	2766	2938	0.87
KGIS-E	Kuder and Zytowski, 1988	Scientific	2	1	1987	1	5894	2714	3180	0.96
KGIS-E	Kuder and Zytowski, 1988	Scientific	2	1	1987	2	7113	3402	3711	0.80
KOIS	Kuder and Zytowski, 1991	Scientific	2	1	1985		3214	1583	1631	0.23
KCS	Zytowski, 2007	Scientific	2	1	2007		3619	1663	1956	0.33
JVIS	Jackson, 2000	Physical Science	2	1	1999	2	2380	1190	1190	0.71
JVIS	Jackson, 2000	Physical Science	2	1	1999	5	1120	560	560	0.52
JVIS	Jackson, 1977	Physical Science	2	1	1977	3	1000	500	500	0.76
GZII	Guilford and Zimmerman, 1989	Scientific	2	1	1989		215	97	118	0.88
**NATURAL SCIENCES**										
CAI-V	Johansson, 1984	Science	2	2	1976	5	1500	750	750	0.24
CISS	Campbell et al., 1992	Science	2	1	1992	5	5241	3442	1799	0.43
IDEAS	Johansson, 1996	Science	2	2	1989	1	1770	820	950	0.65
IDEAS	Johansson, 1996	Science	2	2	1989	2	2891	1208	1683	0.50
IDEAS	Johansson, 1978	Science	2	1	1977	1	598	292	306	0.30
IDEAS	Johansson, 1978	Science	2	1	1977	2	3436	1755	1681	0.35
**BIOLOGICAL SCIENCES**										
JVIS	Jackson, 2000	Life Science	2	1	1999	2	2380	1190	1190	0.24
JVIS	Jackson, 2000	Life Science	2	1	1999	5	1120	560	560	0.14
JVIS	Jackson, 1977	Life Science	2	1	1977	3	1000	500	500	0.18
**MEDICAL SCIENCE**										
SII	Donnay et al., 2005	Medical Science	2	1	2002	5	2250	1125	1125	0.06
SII	Harmon et al., 1994	Medical Science	2	1	1994	5	18,951	9484	9467	0.09
SII	Hansen and Campbell, 1985	Medical Science	2	1	1985	5	600	300	300	0.18
SII	Campbell, 1974	Medical Science	2	1	1974	5	600	300	300	0.01
CAI-E	Johansson, 2003	Medical Science	2	2	1986	5	900	450	450	−0.20
OVIS-II	Winefordner, 1983	Medical Service	2	2	1980	1	9242	4604	4638	−0.23
OVIS-II	Winefordner, 1983	Medical Service	2	2	1980	2	6416	3157	3259	−0.21
OVIS-II	Winefordner, 1983	Medical Service	2	2	1980	3	2792	1055	1737	−0.06
OVIS	D'Costa et al., 1970	Medical	2	1	1969	2	45,845	23,062	22,783	−0.13
IDEAS	Johansson, 1996	Medical	2	2	1989	1	1770	820	950	−0.15
IDEAS	Johansson, 1996	Medical	2	2	1989	2	2891	1208	1683	−0.20
JVIS	Jackson, 2000	Medical Service	2	1	1999	2	2380	1190	1190	0.15
JVIS	Jackson, 2000	Medical Service	2	1	1999	5	1120	560	560	0.11
JVIS	Jackson, 1977	Medical Service	2	1	1977	3	1000	500	500	0.12
VII	Lunneborg, 1981	Science	2	3	1976	2	600	300	300	−0.17
VII-R	Lunneborg, 1993	Science	2	3	1985	2	1562	748	814	−0.03
**MEDICAL SERVICES**										
SII	Donnay et al., 2005	Healthcare Services	1	1	2002	5	2250	1125	1125	−0.24
SII	Harmon et al., 1994	Medical Service	1	1	1994	5	18,951	9484	9467	−0.17
SII	Hansen and Campbell, 1985	Medical Service	1	1	1985	5	600	300	300	−0.04
SII	Campbell, 1974	Medical Service	1	1	1974	5	600	300	300	−0.52
CAI-E	Johansson, 2003	Medical Service	1	2	1986	5	900	450	450	−0.12
CAI-V	Johansson, 1984	Medical Service	1	2	1976	5	1500	750	750	−0.47
CISS	Campbell et al., 1992	Medical Practice	1	1	1992	5	5241	3442	1799	−0.13
OVIS-II	Winefordner, 1983	Health Services	1	2	1980	1	9800	4479	4552	−0.58
OVIS-II	Winefordner, 1983	Health Services	1	2	1980	2	6672	3120	3217	−0.55
OVIS-II	Winefordner, 1983	Health Services	1	2	1980	3	2800	1052	1730	−0.32
OVIS	D'Costa et al., 1970	Nursing and Related Technical Services	1	1	1969	2	46,065	23,203	22,862	−0.90
IDEAS	Johansson, 1978	Medical Service	1	1	1977	1	598	292	306	−0.75
IDEAS	Johansson, 1978	Medical Service	1	1	1977	2	3436	1755	1681	−0.90
CII-1	Psychological Corporation, 1991	Health Services	1	1	1989	1	13,280	6545	6735	−0.42
CII-1	Psychological Corporation, 1991	Health Services	1	1	1989	1	19,780	9825	9955	−0.46
CII-1	Psychological Corporation, 1991	Health Services	1	1	1989	1	26,082	13,123	12,959	−0.50
CII-2	Psychological Corporation, 1991	Health Services	1	1	1989	2	14,300	6987	7313	−0.38
CII-2	Psychological Corporation, 1991	Health Services	1	1	1989	2	8556	4208	4348	−0.35
CII-2	Psychological Corporation, 1991	Health Services	1	1	1989	2	8399	4051	4348	−0.36
CDI	Jackson, 1986	Health Services	1	1	1986	3	1000	500	500	−0.37
CDI	Jackson, 2003	Health Services	1	1	2003	1	212	114	98	−0.43
CDI	Jackson, 2003	Health Services	1	1	2003	2	737	385	352	−0.34
CDI	Jackson, 2003	Health Services	1	1	2003	3	386	206	180	−0.31
CDI	Jackson, 2003	Health Services	1	1	2003	4	392	171	221	−0.30
CDI	Jackson, 2003	Health Services	1	1	2003	5	317	148	169	0.00
CDI	Jackson, 2003	Health Services	1	1	2003	5	276	145	131	−0.32
**SOCIAL SCIENCES**										
SII	Donnay et al., 2005	Social Sciences	2	1	2002	5	2250	1125	1125	−0.08
CII-1	Psychological Corporation, 1991	Social Science	2	1	1989	1	13,190	6491	6699	−0.42
CII-1	Psychological Corporation, 1991	Social Science	2	1	1989	1	19,674	9757	9917	−0.50
CII-1	Psychological Corporation, 1991	Social Science	2	1	1989	1	26,009	13,056	12,953	−0.59
CII-2	Psychological Corporation, 1991	Social Science	2	1	1989	2	14,871	7346	7525	−0.44
CII-2	Psychological Corporation, 1991	Social Science	2	1	1989	2	8849	4392	4457	−0.37
CII-2	Psychological Corporation, 1991	Social Science	2	1	1989	2	8679	4241	4438	−0.40
JVIS	Jackson, 2000	Social Science	2	1	1999	2	2380	1190	1190	−0.08
JVIS	Jackson, 2000	Social Science	2	1	1999	5	1120	560	560	−0.10
JVIS	Jackson, 1977	Social Science	2	1	1977	3	1000	500	500	−0.33
**SCIENCE TECHNICIANS**										
COPS-R	Knapp and Knapp, 1979	Science-Skilled	1	3	1979	2	400	200	200	0.02
COPS	Knapp et al., 1990	Science-Skilled	1	1	1988	2	14,619	7565	7054	0.13
COPS	Knapp et al., 1990	Science-Skilled	1	1	1988	3	3237	1379	1858	0.12
COPS	Knapp and Knapp, 1984	Science-Skilled	1	1	1982	2	4145	2034	2111	0.24
COPS	Knapp and Knapp, 1984	Science-Skilled	1	1	1982	3	1445	773	672	0.17
**ENGINEERING**										
OVIS-II	Winefordner, 1983	Engineering and Physical Science	2	2	1980	1	9119	4530	4589	0.86
OVIS-II	Winefordner, 1983	Engineering and Physical Science	2	2	1980	2	6368	3132	3236	0.81
OVIS-II	Winefordner, 1983	Engineering and Physical Science	2	2	1980	3	2781	1052	1729	0.90
OVIS	D'Costa et al., 1970	Applied Technology	2	1	1969	2	45,832	23,058	22,774	1.55
CII-1	Psychological Corporation, 1991	Mathematics and Science	2	1	1989	1	13,190	6491	6699	0.48
CII-1	Psychological Corporation, 1991	Mathematics and Science	2	1	1989	1	19,674	9757	9917	0.61
CII-1	Psychological Corporation, 1991	Mathematics and Science	2	1	1989	1	26,009	13,056	12,953	0.68
CII-2	Psychological Corporation, 1991	Mathematics and Science	2	1	1989	2	14,871	7346	7525	0.48
CII-2	Psychological Corporation, 1991	Mathematics and Science	2	1	1989	2	8849	4392	4457	0.46
CII-2	Psychological Corporation, 1991	Mathematics and Science	2	1	1989	2	8679	4241	4438	0.43
JVIS	Jackson, 2000	Engineering	2	1	1999	2	2380	1190	1190	1.24
JVIS	Jackson, 2000	Engineering	2	1	1999	5	1120	560	560	0.85
JVIS	Jackson, 1977	Engineering	2	1	1977	3	1000	500	500	1.13
VII	Lunneborg, 1981	Technical	2	3	1976	2	600	300	300	0.95
VII-R	Lunneborg, 1993	Technical	2	3	1985	2	1562	748	814	0.52
COPS-R	Knapp and Knapp, 1979	Technology-Professional	2	3	1979	2	400	200	200	0.19
COPS	Knapp et al., 1990	Technology-Professional	2	1	1988	2	14,619	7565	7054	1.01
COPS	Knapp et al., 1990	Technology-Professional	2	1	1988	3	3237	1379	1858	0.89
COPS	Knapp and Knapp, 1984	Technology-Professional	2	1	1982	2	4145	2034	2111	1.05
COPS	Knapp and Knapp, 1984	Technology-Professional	2	1	1982	3	1445	773	672	0.96
GZII	Guilford and Zimmerman, 1989	Mechanical	2	1	1989		215	97	118	1.28
WOWI	Ripley et al., 2001	Engineering and Related	2	1	1997		169,436	78,564	90,872	0.95
**ENGINEERING TECHNICIAN**										
OVIS	D'Costa et al., 1970	Appraisal	1	1	1969	2	46,002	23,151	22,851	1.19
CDI	Jackson, 1986	Science and Technology	1	1	1986	3	1000	500	500	0.98
CDI	Jackson, 2003	Science	1	1	2003	1	212	114	98	0.96
CDI	Jackson, 2003	Science	1	1	2003	2	737	385	352	0.74
CDI	Jackson, 2003	Science	1	1	2003	3	386	206	180	0.71
CDI	Jackson, 2003	Science	1	1	2003	4	392	171	221	0.87
CDI	Jackson, 2003	Science	1	1	2003	5	317	148	169	0.93
CDI	Jackson, 2003	Science	1	1	2003	5	276	145	131	0.75
**MECHANICS AND ELECTRONICS**										
SII	Donnay et al., 2005	Mechanics and Construction	1	1	2002	5	2250	1125	1125	1.02
SII	Donnay et al., 2005	Computer Hardware and Electronics	1	1	2002	5	2250	1125	1125	0.77
SII	Harmon et al., 1994	Mechanical Activities	1	1	1994	5	18,951	9484	9467	0.68
SII	Hansen and Campbell, 1985	Mechanical Activities	1	1	1985	5	600	300	300	0.70
SII	Campbell, 1974	Mechanical	1	1	1974	5	600	300	300	0.91
CAI-E	Johansson, 2003	Mechanical/Fixing	1	2	1986	5	900	450	450	0.54
CAI-E	Johansson, 2003	Electronics	1	2	1986	5	900	450	450	0.63
CAI-V	Johansson, 1984	Mechanical/Fixing	1	2	1976	5	1500	750	750	1.15
CAI-V	Johansson, 1984	Electronics	1	2	1976	5	1500	750	750	1.15
CISS	Campbell et al., 1992	Mechanical Crafts	1	1	1992	5	5241	3442	1799	1.02
IDEAS	Johansson, 1996	Mechanical/Fixing	1	2	1989	1	1770	820	950	1.15
IDEAS	Johansson, 1996	Mechanical/Fixing	1	2	1989	2	2891	1208	1683	1.15
IDEAS	Johansson, 1978	Mechanical/Fixing	1	1	1977	1	598	292	306	1.15
IDEAS	Johansson, 1978	Mechanical/Fixing	1	1	1977	2	3436	1755	1681	1.15
IDEAS	Johansson, 1978	Electronics	1	1	1977	1	598	292	306	1.15
IDEAS	Johansson, 1978	Electronics	1	1	1977	2	3436	1755	1681	1.20
KGIS-E	Kuder, 1964	Mechanical	1	1	1963	1	4109	2080	2029	2.12
KGIS-E	Kuder, 1964	Mechanical	1	1	1963	2	5704	2766	2938	1.83
KGIS-E	Kuder and Zytowski, 1988	Mechanical	1	1	1987	1	5894	2714	3180	1.86
KGIS-E	Kuder and Zytowski, 1988	Mechanical	1	1	1987	2	7113	3402	3711	1.91
KOIS	Kuder and Zytowski, 1991	Mechanical	1	1	1985		3214	1583	1631	1.14
KCS	Zytowski, 2007	Mechanical	1	1	2007		3619	1663	1956	0.97
CDI	Jackson, 1986	Industrial Arts	1	1	1986	3	1000	500	500	1.31
CDI	Jackson, 2003	Industrial Arts	1	1	2003	1	212	114	98	2.21
CDI	Jackson, 2003	Industrial Arts	1	1	2003	2	737	385	352	1.77
CDI	Jackson, 2003	Industrial Arts	1	1	2003	3	386	206	180	1.40
CDI	Jackson, 2003	Industrial Arts	1	1	2003	4	392	171	221	1.06
CDI	Jackson, 2003	Industrial Arts	1	1	2003	5	317	148	169	0.98
CDI	Jackson, 2003	Industrial Arts	1	1	2003	5	276	145	131	1.26
OASIS:IS	Parker, 2002	Mechanical	1	2	1991	2	1091	551	540	1.25
CCQ-S	Chronicle Guidance Publications, 1992	Mechanical	1	2	1990	1	1536	797	739	1.08
CCQ-L	Chronicle Guidance Publications, 1992	Mechanical	1	2	1990	2	1311	661	650	1.08
GOCL II	Gordon, 1981	Technology–Mechanical	1	1	1981	2	359	168	191	0.62
VRII	Vocational Research Institute, 1988	Mechanical	1	1	1985	2	856	429	427	1.51
VRII	Vocational Research Institute, 1988	Mechanical	1	1	1985	4	525	198	327	0.71
WOWI	Ripley et al., 2001	Mechanical and Electrical Work	1	1	1997		169,436	78,564	90,872	1.07
**COMPUTER SCIENCE**										
SII	Donnay et al., 2005	Programming and Information System	2	1	2002	5	2250	1125	1125	0.38
**MATHEMATICS**										
SII	Donnay et al., 2005	Mathematics	2	1	2002	5	2250	1125	1125	0.46
SII	Harmon et al., 1994	Mathematics	2	1	1994	5	18,951	9484	9467	0.24
SII	Hansen and Campbell, 1985	Mathematics	2	1	1985	5	600	300	300	0.26
SII	Campbell, 1974	Mathematics	2	1	1974	5	600	300	300	0.35
CAI-E	Johansson, 2003	Mathematics	2	2	1986	5	900	450	450	0.22
CISS	Campbell et al., 1992	Mathematics	2	1	1992	5	5241	3442	1799	0.35
JVIS	Jackson, 2000	Mathematics	2	1	1999	2	2380	1190	1190	0.55
JVIS	Jackson, 2000	Mathematics	2	1	1999	5	1120	560	560	0.45
JVIS	Jackson, 1977	Mathematics	2	1	1977	3	1000	500	500	0.55
**APPLIED MATHEMATICS**										
SII	Harmon et al., 1994	Data Management	1	1	1994	5	18,951	9484	9467	0.16
CAI-V	Johansson, 1984	Numbers	1	2	1976	5	1500	750	750	0.05
OVIS-II	Winefordner, 1983	Numerical	1	2	1980	1	8917	4425	4492	0.29
OVIS-II	Winefordner, 1983	Numerical	1	2	1980	2	6315	3109	3206	0.27
OVIS-II	Winefordner, 1983	Numerical	1	2	1980	3	2780	1052	1728	0.62
OVIS	D'Costa et al., 1970	Numerical	1	1	1969	2	46,015	23,164	22,851	0.52
IDEAS	Johansson, 1996	Mathematics	1	2	1989	1	1770	820	950	0.05
IDEAS	Johansson, 1996	Mathematics	1	2	1989	2	2891	1208	1683	0.00
IDEAS	Johansson, 1978	Numbers	1	1	1977	1	598	292	306	0.00
IDEAS	Johansson, 1978	Numbers	1	1	1977	2	3436	1755	1681	0.05
KGIS-E	Kuder, 1964	Computational	1	1	1963	1	4109	2080	2029	0.30
KGIS-E	Kuder, 1964	Computational	1	1	1963	2	5704	2766	2938	0.44
KGIS-E	Kuder and Zytowski, 1988	Computational	1	1	1987	1	5894	2714	3180	0.18
KGIS-E	Kuder and Zytowski, 1988	Computational	1	1	1987	2	7113	3402	3711	0.27
KOIS	Kuder and Zytowski, 1991	Computational	1	1	1985		3214	1583	1631	0.27
KCS	Zytowski, 2007	Computational	1	1	2007		3619	1663	1956	0.20

*d, inverse variance weighted effect size; CAI-E, Career Assessment Inventory–Enhanced Version; CAI-V, Career Assessment Inventory–Vocational Version; CCQ-S, Chronicle Career Quest (Form S); CCQ-L, Chronicle Career Quest (Form L); CDI, Career Decision Inventory; CII-1, Career Interest Inventory (Level 1); CII-2, Career Interest Inventory (Level 2); CISS, Campbell Interest and Skill Survey; COPS, Career Occupational Preference System Interest Inventory; COPS-R, Career Occupational Preference System Interest Inventory–Revised; GOCL II, Gordon Occupational Check List II; GZII, Guilford–Zimmerman Interest Inventory; IDEAS, Interest Determination, Exploration and Assessment System; JVIS, Jackson Vocational Interest Survey; KGIS-E, Kuder General Interest Survey (Form E); KOIS, Kuder Occupational Interest Survey; KCS, Kuder Career Search with Person Match; OASIS:IS, Occupational Aptitude Survey and Interest Schedule: Interest Schedule; OVIS, Ohio Vocational Interest Survey; SII, Strong Interest Inventory; VII, Vocational Interest Inventory; VII-R, Vocational Interest Inventory–Revised; VRII, Vocational Research Interest Inventory; WOWI, World of Work Inventory. In the coding for job complexity, 1, technical level, 2, professional level. For item development strategy (Gender_balanced), 1 represents an overlap of male and female scores of less than 75% or cases in which more than 33% of the items have response differences larger than 15%; 2 represents an overlap of male and female scores from 75 to 85% or 10 to 33% of the items have response differences larger than 15%; 3 represents an overlap of male and female scores larger than 85% or in which no more than 10% of the items have response differences larger than 15%. Age group was coded as the following: 1, middle school students or 12–14 years old; 2, high school students or 15–18 years old; 3, college students or 19–22 years old; 4, emerging working adults or 23–30 years old; 5, experienced working adults or 31 years and older*.

### Identification of occupational characteristics and statistics

We obtained occupational-level information from two sources: information about the people-orientation, things-orientation, and level of required quantitative ability was acquired through the O^*^NET production database 18.1 (National Center for O^*^NET Development, 2014), and information about the percentages of women in STEM fields was obtained from the U.S. Bureau of Labor Statistics (2014) latest report *Women in the Labor Force: A Databook*. Both sources used the 2010 Standard Occupational Classification (SOC; U.S. Bureau of Labor Statistics, 2010) system, allowing us to combine two sources of information using matching occupational codes.

The O^*^NET database provides comprehensive and regularly updated information on various aspects of worker attributes and job requirements for over 900 U.S. occupations, including occupational interest profiles (OIPs; Rounds et al., 1999) and levels of required abilities (McCoy et al., 1999; Donsbach et al., 2003). The OIPs are organized using Holland's (1997) RIASEC typology for describing work environments. The scores on each OIP indicate how well the occupation represents the six types of work environments. For example, the *Realistic* score for an occupation indicates how characteristic the occupation is of a things-oriented work environment; the *Social* score for an occupation indicates how descriptive the occupation is of a people-oriented work environment. Therefore, the *Realistic* and *Social* scores on the OIPs were used to represent the things- and people-orientations for an occupation, respectively. Both are on a scale from 1 to 7, with higher scores indicating stronger things- or people-orientation. The O^*^NET system includes scores on two types of quantitative ability required by each occupation: Mathematical Reasoning (i.e., the ability to choose the right mathematical methods or formulas to solve a problem) and Number Facility (i.e., the ability to add, subtract, multiply, or divide quickly and correctly). Because scores for the two types of quantitative ability are highly correlated (*r* > 0.90), in the current study the average score was taken to represent the level of quantitative ability required by each occupation. The score for required level of quantitative ability ranged from 0 to 6, with higher scores indicating greater ability required.

The occupational-level characteristics and statistics were then aggregated to each of the 13 STEM fields following the SOC system. For example, the people-orientation, things-orientation, and required level of quantitative ability for *Physical Sciences* were calculated by averaging the information from all the occupations nested within it, including Astronomers and Physicists, Atmospheric and Space Scientists, Chemists and Materials Scientists, Environmental Scientists, and Geoscientists. The percentage of women in *Physical Sciences* was calculated by dividing the total number of females employed in the above occupations by the total number of males and females employed. For *Mathematics*, the occupational characteristics and statistics were calculated from the data for Mathematicians and Statisticians. Similar calculations were performed for the rest of the STEM fields.

### Coding of additional moderators

As discussed previously, we coded the complexity of the activities measured by a basic interest scale (professional-level = 2, technical-level = 1). The age group of a sample was coded based on the sample description and mean age of the sample reported in an interest inventory technical manual (middle school students or 12–14 years old = 1, high school students or 15–18 years old = 2, college students or 19–22 years old = 3, emerging working adults or 23–30 years old = 4, and experienced working adults or 31 years and older = 5). The years of data collection were also identified from the interest inventory technical manuals, ranging from 1963 to 2007. Information on item development strategy, or the degree to which an interest inventory was developed to be gender-balanced, was obtained from Su et al. (2009) and was coded as the following: overlap of male and female interest scores was less than 75% or more than 33% of the items had response differences larger than 15% = 1; overlap of male and female scores was between 75 and 85% or 10 to 33% of the items had response differences larger than 15% = 2; overlap of male and female scores was larger than 85% or no more than 10% of the items had response differences larger than 15% = 3. Coding for these additional moderators, along with the sample sizes by gender and total sample size for each sample, are listed in Table 1.

### Analytical procedures

To examine gender differences in basic interests across STEM fields, we first calculated the standardized mean difference between males and females (Cohen's *d*) for each basic interest scale. This step yielded a total of 173 effect sizes, presented in Table 1. In the case where an interest inventory had more than one basic interest scales assessing a STEM field (e.g., both a *Mechanics* scale and an *Electronics* scale for the field of Mechanics and Electronics), we averaged the effect sizes within sample to avoid statistical dependence, creating 168 independent effect sizes. We then followed the procedures outlined in Hedges and Olkin (1985) and Lipsey and Wilson (2001) to calculate the standard error and inverse-variance weight for each effect size, correct the effect sizes for small-sample-size bias, and synthesize the effect sizes. As discussed previously, we expected to find heterogeneity among the effect sizes. Instead of focusing on the grand mean gender difference in interests across all STEM fields, the main goal of our study was to understand how the average gender difference in interests varies by STEM field. Therefore, we conducted a meta-analytic analog of (inverse-variance weighted) analysis of variance (ANOVA) to compare the average gender differences in interests in different STEM fields, using a mixed-effects model (cf. Viechtbauer, 2008, for the rationale to start with the mixed-effects model for meta-analyses that are focusing on moderators).

Next, to understand the occupational characteristics associated with gender differences in interests and other variables that potentially moderate the effect sizes, we conducted a inverse-variance weighted meta-regression to evaluate the effects of the people-orientation, things-orientation, required level of quantitative ability, and job complexity (professional- vs. technical-level) of a STEM field as well as the age group of a sample and the year of data collection, again using a mixed-effects model. The weighted ANOVA and weighted meta-regression analysis were both performed using the statistical macros provided by Wilson (2005).

To examine the relationship between the gender differences in interests and gender composition within STEM fields, we conducted correlation and regression analyses at the occupational level, using occupational characteristics and statistics aggregated to the 13 STEM fields.

## Results

As expected, we found gender differences in interests to be heterogeneous and to vary largely across the 13 STEM fields. We summarized the effect sizes of gender differences in interests by STEM field in Table 2. In addition to the weighted mean effect size, *d*, we reported *k*, the number of effect sizes used to compute each mean effect size, *N*, the number of total respondents within a STEM field, as well as the 95% confidence interval and 90% credibility values for each mean effect size^2^. A positive *d* value indicates that men had stronger interests in the STEM field than women and a negative *d*-value indicates stronger interests for women.

**Table 2:** **Weighted mean effect sizes and distribution of heterogeneity by STEM field**.

**Basic interest scale**	***k***	***N***	***d***	***SE***	**Lower 95%CI**	**Upper 95%CI**	**Lower 90%CV**	**Upper 90%CV**	***Q_W_***	***p***
Physical Sciences	15	57,669	**0.56**	0.06	**0.43**	**0.69**	**0.09**	**1.03**	16.84	0.26
Natural Sciences	6	15,436	**0.41**	0.11	**0.21**	**0.62**	**0.17**	**0.66**	1.66	0.89
Biological Science	3	4,500	0.19	0.15	−0.11	0.48	**0.10**	**0.27**	0.08	0.96
Medical Science	16	98,919	−0.04	0.06	−0.17	0.08	−0.28	0.20	4.74	0.99
Social Sciences	10	98,022	**−0.33**	0.08	**−0.49**	**−0.17**	**−0.63**	**−0.03**	4.62	0.87
Science Technicians	5	23,846	0.14	0.12	−0.09	0.37	**0.01**	**0.27**	0.37	0.98
Medical Services	26	193,130	**−0.40**	0.05	**−0.50**	**−0.30**	**−0.77**	**−0.03**	18.84	0.80
Engineering	22	355,531	**0.83**	0.06	**0.72**	**0.94**	**0.29**	**1.36**	**32.88**	**0.05**
Engineering Technicians	8	49,322	**0.89**	0.10	**0.71**	**1.08**	**0.64**	**1.15**	2.59	0.92
Mechanics and Electronics	31	255,508	**1.21**	0.05	**1.12**	**1.30**	**0.50**	**1.92**	**78.15**	**0.00**
Computer Science	1	2,250	0.38	−	−	−	−	−	−	−
Mathematics	9	33,042	**0.38**	0.09	**0.21**	**0.55**	**0.17**	**0.59**	1.95	0.98
Applied Mathematics	26	122,826	**0.23**	0.06	**0.11**	**0.35**	−0.07	0.53	7.70	0.94
				**Within-field heterogeneity (*Q_W_*):**				170.42 (*df* = 155)		0.19
				**Between-field heterogeneity (*Q_B_*):**				**776.45** (*df* = 12)		**0.00**
				**Total heterogeneity (*Q_T_*):**				**946.88** (*df* = 167)		**0.00**

*k, Number of effect sizes; N, number of respondents; d, inverse variance weighted effect sizes, a positive d-value indicates gender difference favoring men and a negative d-value indicates gender difference favoring women; SE, standard error for d; CI, confidence interval; CV, credibility value; Q, heterogeneity statistic; p, probability of significance value associated with the Q statistic; bolded confidence intervals and credibility values indicate that 0 is not included within the interval; bolded Q statistic and corresponding p-value indicate that there was significant total heterogeneity between studies and significant heterogeneity among the effect sizes across STEM fields*.

The most notable finding was that gender differences in interests varied greatly by STEM field: the largest gender differences in interests were observed in Engineering disciplines (*d* = 0.83, 0.89, and 1.21 for Engineering—professional level, Engineering Technicians, and Mechanics and Electronics, respectively), favoring men. In contrast, no significant gender differences in interests were found in Biological and Medical sciences, neither in the technical aspects of scientific activities. In Social Sciences and Medical Services, arguably the most people-oriented fields, women exhibited stronger interests than men (*d* = −0.33 and −0.40, respectively). Importantly, results from inverse-variance weighted ANOVA showed that the majority of heterogeneity among the effect sizes was introduced by dissimilarities between STEM fields (*Q_B_* = 776.45, *df* = 12, *p* < 0.001), rather than from within STEM fields (*Qw* = 170.42, *df* = 155, *p* = 0.19). The observed gender differences in interests within each STEM field were homogeneous for 11 of the 13 STEM fields. Only two exceptions—Engineering, and Mechanics and Electronics—had significant within-field variations, with effect sizes ranging 0.19 to 1.55 for Engineering and from 0.54 to 2.21 for Mechanics and Electronics.

Table 3 presents findings from the meta-regression on the effects of covariates of gender differences in interests, including the people-orientation, things-orientation, level of quantitative ability required, and job complexity (professional- vs. technical-level) of a STEM field as well as the age group of a sample and year of data collection. Consistent with our hypotheses 1a and 1b, gender differences of interests in various STEM fields can be explained by the people-orientation and things-orientation of the disciplines. The size of gender differences in interests (favoring men) increased with higher things-orientation of a STEM field (*B* = 0.18, β = 0.48, *p* < 0.001) and decreased with higher people-orientation (*B* = −0.19, β = −0.60, *p* < 0.001). In contrast, the level of quantitative ability required did not predict differential interests of men and women in a STEM field (*B* = 0.02, β = 0.03, *p* = 0.68). Hypothesis 2 was also supported. Job complexity and gender-balanced item development strategy each had a small effect (smaller gender differences in interests at the professional level compared to the technical level, *B* = −0.10, β = −0.08, *p* = 0.11, and small gender differences in interests with more aggressive gender-balanced item development strategy, *B* = −0.08, β = −0.07, *p* = 0.08), yet neither was significant at the *p* < 0.05 level. The age group of a sample and the year of data collection did not influence the size of gender differences in interests. The meta-regression model (primarily people- and things-orientations) explained 76.98% of the total between-study heterogeneity (*Q*_M_ = 532.87, *df* = 7, *p* < 0.001) and the residual heterogeneity was not significant (*Q_E_* = 159.37, *df* = 150, *p* = 0.28), indicating that people-orientation and things-orientation of the STEM fields were the main contributors to the variation in effect sizes across STEM fields.

**Table 3:** **Meta regression coefficients for covariates of gender differences in STEM interests**.

**Model**	***B***	***SE***	**Lower 95%CI**	**Upper 95%CI**	***Z***	***β***	***p***
Constant	3.46	4.815	−5.98	12.90	0.72	0.00	0.47
Things-orientation	**0.18**	0.024	**0.14**	**0.23**	7.65	**0.48**	**0.00**
People-orientation	**−0.19**	0.018	**−0.22**	**−0.15**	−10.67	−**0.60**	**0.00**
Required level of quantitative ability	0.02	0.053	−0.08	0.13	0.41	0.03	0.68
Job complexity	−0.10	0.062	−0.22	0.02	−1.60	−0.08	0.11
Gender-balanced item development strategy	−0.08	0.048	−0.18	0.01	−1.76	−0.07	0.08
Year of data collection	−0.00	0.002	−0.01	0.00	−0.66	−0.03	0.51
Age group of sample	−0.02	0.017	−0.05	0.02	−1.02	−0.04	0.31
**Model explained heterogeneity (*Q_M_*):**					**532.87** (*df* = 7)		**0.00**
**Residual heterogeneity (*Q_E_*):**					159.37 (*df* = 150)		0.28
**Total heterogeneity (*Q_T_*):**					**692.24** (*df* = 157)		**0.00**
**R2 analog (amount of heterogeneity accounted for):**							**76.98%**

*B, unstandardized regression coefficient; SE, standard error for B; CI, confidence interval; Z, standard score for B; β, standardized regression coefficient; p, probability of significance value for regression coefficients; bolded confidence intervals indicate that 0 is not included within the interval; bolded Qstatistic and corresponding p-value indicate that total heterogeneity between studies was significant and the model explained a significant amount of heterogeneity*.

Finally, we looked at the gender composition in STEM occupations and examined its association with gender differences in interests and various occupational characteristics. In Table 4, we report the percentage of women by STEM field, along with the level of quantitative ability required, things-, and people-orientations for each field. We again present the effect size of gender difference in interests (*d*) for each STEM field and report two additional statistics^3^ associated with *d*: (1) We calculated the percentage of overlap (Bhattacharyya coefficient) between male and female interest distributions given the effect size of gender difference in interests for each STEM field. This statistic provides an additional, intuitive metric to represent the similarity and dissimilarity of men's and women's interests. A higher percentage of overlap indicates more similar interests between men and women, and a lower percentage of overlap indicates more dissimilar interests. (2) We calculated the percentage of women within the top 10% of the total population in the interest distribution. This statistic provides an index on how well women are represented among those who are most strongly interested in a STEM field. Assuming that individuals at the right tail (highest 10%) of a population interest distribution are likely to choose a career in that basic interest area (e.g., *Mathematics*), this statistic also represents the hypothetical/projected percentage of women who would work in a STEM field given the gender difference in interests. These statistics can provide further insight into men and women's differential interests in various STEM fields and a more straightforward comparison with the actual gender distribution in each field.

**Table 4:** **Occupational characteristics, gender differences in interests, and percentage of females by STEM field**.

**STEM Field**	**Quantitative ability**	**Things-orientation**	**People-orientation**	**M–F *d* in interests**	**M–F interest overlap (%)**	***p(F)* in top 10% interests (%)**	***p(F)* in STEM field (%)**
Physical Sciences	4.14	4.89	1.67	0.56	77.84	35.79	32.80
Natural Sciences	4.00	4.82	1.64	0.41	83.64	39.47	35.41
Biological Science	3.68	4.67	1.58	0.19	92.54	45.18	45.74
Medical Science	3.07	4.61	5.13	−0.04	98.30	51.10	43.13
Social Sciences	3.09	2.22	3.39	−0.33	86.76	58.53	58.46
Science Technicians	3.16	5.27	1.76	0.14	94.47	46.45	44.66
Medical Services	2.59	4.02	6.41	−0.40	84.25	60.14	89.41
Engineering	3.97	5.75	1.46	0.83	67.91	29.61	10.98
Engineering Technicians	3.27	6.26	1.11	0.89	65.46	28.12	12.18
Mechanics and Electronics	2.09	6.95	1.23	1.21	54.47	21.61	2.91
Computer Science	3.33	4.51	1.76	0.38^*^	84.93	40.30	23.75
Mathematics	5.44	2.00	1.00	0.38	84.86	40.25	39.58
Applied Mathematics	4.21	1.97	1.86	0.23	90.80	44.06	58.57

* *Estimated based on one interest inventory. M–F, Male–Female; d, inverse variance weighted effect sizes, a positive d-value indicates gender difference favoring men and a negative d-value indicates gender difference favoring women; p(F), percentage of females*.

Table 5 presents the correlations among occupational characteristics, gender differences in interests, and the percentages of women across STEM fields. As expected, people-orientation and things-orientation were associated with the percentage of women in a STEM field (*r* = 0.72, *p* < 0.01, and *r* = −0.66, *p* < 0.05, respectively). The percentages of women were higher in STEM fields that are more people-oriented and less things-oriented. The percentages of women in STEM fields were also very strongly correlated with gender differences in interests (*r* = −0.89, *p* < 0.01). The percentages of women were higher in STEM fields in which men and women were more equally interested or those for which women had stronger interests.

**Table 5:** **Correlations among occupational characteristics, gender differences in interests, and percentages of females in STEM fields**.

	**Quantitative ability**	**People-orientation**	**Things-orientation**	**M–F *d* in interests**
People-orientation	−0.44			
Things-orientation	−0.58	−0.21		
M–F *d* in interests	0.00	−0.73^**^	0.65^*^	
Percentage of females	−0.00	0.72^**^	−0.66^*^	−0.89^**^

** Correlation is significant at the 0.01 level;

* *Correlation is significant at the 0.05 level*.

Further, hierarchical regression analysis showed that, after controlling for the effect of gender differences in interests, the effect of people-orientation decreased substantially and was no longer significant (β = 0.14, *p* = 0.50 for people-orientation; β = −0.79, *p* < 0.01 for gender differences in interests). Similarly, after controlling for the effect of gender differences in interests, the effect of things-orientation decreased substantially and was no longer significant (β = −0.14, *p* = 0.47 for things-orientation; β = −0.80, *p* < 0.01 for gender differences in interests). These results indicated that the effects of people- and things-orientations on the gender composition (percentage of women) in STEM fields were mediated through the differential interests of men and women. Hypotheses 3, 4a, and 4b were supported. Consistent with Hypothesis 5, the percentage of women in a STEM field was not associated with the level of quantitative ability required by the field.

**Figure 1:**
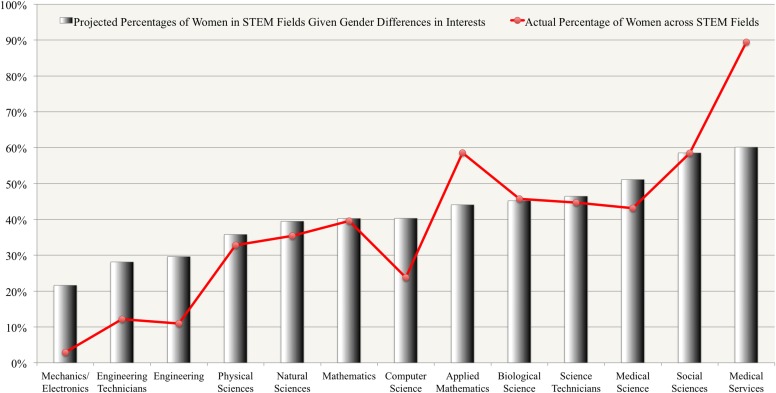
**Comparison between projected percentages of women in STEM fields given gender differences in interests and actual gender composition across STEM fields**.

To visualize the relationship between gender differences in interests and the gender composition across STEM fields, we plotted the projected percentages of women given the gender differences in interests in comparison with the actual percentages of women in various STEM fields in Figure 1. As shown in Figure 1, the actual percentages of women closely mirror the projected percentages of women given the gender differences in interests in *Mathematics* and the sciences (*Physical Sciences, Natural Sciences, Biological Science, Medical Science, Social Sciences*, and *Science-Technicians*). However, the actual percentages of women fell short of the predicted percentages based on interests in the Engineering disciplines (*Engineering, Engineering-Technicians, Mechanics and Electronics*, and *Computer Science*). The percentages of women exceeded the predicted percentages based on interests in *Applied Mathematics* and *Medical Services*. These results suggest that men and women's participation in these fields were potentially influenced by factors other than interests.

## Discussion

Increasing the representation of women in the STEM workforce poses one of the most critical challenges for our society. To date, research to understand gender disparities in STEM careers typically treated all the STEM fields as a whole and emphasized the similarities among STEM fields rather than their dissimilarities. We argue that STEM fields are heterogeneous. Understanding men's and women's career choices across different STEM fields is as meaningful as understanding the career choices between STEM and non-STEM fields. Therefore, we examined gender differences in basic interests across different STEM fields.

We found drastically different levels of gender differences in basic interests within STEM fields. Large to very large gender differences in interests favoring men were observed in engineering-related fields (*d* = 0.83 for *Engineering*—professional level, *d* = 0.89 for *Engineering Technicians*, and *d* = 1.21 for *Mechanics and Electronics*). Small to moderate gender differences in interests favoring men were observed for mathematical careers (*d* = 0.38 for *Mathematics*, and *d* = 0.23 for *Applied Mathematics*). Gender differences in interests vary largely in the sciences, ranging from moderate, favoring men, in *Physical Sciences* (*d* = 0.56), to non-significant (*d* = 0.19 for *Biological Science, d* = 0.14 for *Science Technicians*, and *d* = −0.04 for *Medical Science*), and to small to moderate, favoring women (*d* = −0.33 for *Social Sciences*, and *d* = −0.40 for *Medical Services*). These findings provide refined information about men and women's interests in sub-disciplines of STEM. Measuring interests at the basic interest level can produce tailored results about career preferences and can facilitate career guidance for individuals in choosing a STEM career that best matches their interests. Researchers may also gain a clearer understanding of the relationship between interests and career choices by using basic interest measures.

Through investigating gender differences in basic interests across various STEM fields and the occupational characteristics associated with these gender differences in interests, we offer a preference-based explanation for why women are underrepresented in some STEM fields, but not others. Specifically, we argue that individuals' interests are powerful predictors of their occupational membership. Individuals are oriented toward work environments that are congruent with their interests. Men's and women's differences in basic interests lead to unbalanced gender composition in different sectors of the world of work. Two interest dimensions—*Realistic* interests (interest in working with things and gadgets) and *Social* interests (interest in working with people and helping people) may be the most salient in characterizing men and women's differential career preferences, with men having substantially stronger interests in working with things and women preferring working with people. As such, there tend to be larger gender differences in interests (favoring men) for more things-oriented and less people-oriented occupational fields. Overall, STEM fields tend to be high in things-orientation and low in people-orientation. As a result, women on average are less likely to be interested in STEM fields than men, which translate to the lower percentages of women in the STEM workforce. Nonetheless, because STEM disciplines also vary in their things- and people-orientation, women tend to gravitate toward more people-oriented fields within STEM, such as *Medical Science* and *Social Sciences*, as a function of higher *Social* interests.

The current study found the percentages of women within most STEM fields to mirror the gender differences in basic interests in those fields, lending support to the preference-based explanation for gender disparities in STEM careers. Although the projected percentages of females in STEM fields based on interests are only approximations, they provide useful yardsticks for comparing different STEM fields. Information from Figure 1 allows us to identify sub-disciplines of STEM where the shortages of women reflect gender differences in interests and other sub-disciplines where the underrepresentation of women exhibits unexpected patterns. For example, in mathematics and sciences, the actual gender composition is closely aligned with gender differences in interests; however, there are discrepancies between the projected percentages of women based on interests and the actual gender composition in the engineering-related fields and *Medical Services*. The actual percentages of women in engineering-related fields (10.98% for *Engineering*—professional level, 12.18% for *Engineering Technicians*, merely 2.91% for *Mechanics and Electronics*) are even lower than what would be expected based on women's lower interests than men (29.61, 28.12, and 21.61%, respectively). In contrast, the actual percentage of women in *Medical Services* (89.41%) largely exceeded what would be expected based on women's higher interests in this field (60.14%). These results indicate the existence of other factors that escalated the gender disparities in these STEM careers. A few potential factors suggested by the literature include preference for work-life balance (e.g., Ferriman et al., 2009), gender stereotyping and gender role schema in individuals' career decision-making (e.g., Konrad et al., 2000), and implicit bias in employers' selection process (e.g., Moss-Racusin et al., 2012). It is beyond the scope of this article to provide a detailed review of these alternative factors contributing to the gender disparities in the STEM fields (for a comprehensive review, see Ceci et al., 2014). However, the current study points out specific STEM fields where attention to these alternative influences may be most fruitful.

Despite the importance of quantitative ability for STEM careers, we showed that the level of quantitative ability required by a STEM discipline was not associated with men and women's differential interests and representation in that field. To clarify, this result does not mean that quantitative ability is not a consideration in STEM career choices. Instead, it means that the consideration of quantitative ability *at the occupational level* is *equally* important for men and women when choosing a STEM career. *At the individual level*, existing literature (e.g., Lubinski et al., 2001; Wai et al., 2010) has shown that individuals with higher quantitative ability, regardless of their gender, are interested in activities and work environments that require higher levels of quantitative ability and are more likely to choose an occupational field with higher quantitative ability requirement, such as the STEM fields. Individuals with lower quantitative ability, regardless of their gender, are not prepared for entering STEM careers.

Earlier in this article, we discussed the dynamic and reciprocal relationship between interests, knowledge acquisition, and ability development. As previously noted, interests serve as a source of intrinsic motivation for individuals to engage in the activities that they like and accumulate knowledge and skills associated with these activities. Therefore, individuals' interests at an early age may have a profound influence on their ability development through directing the learning process. For example, a girl who is interested in people-oriented activities may choose to focus on classes and extracurricular activities that fulfill her people interests, such as social studies and volunteering at an animal shelter, and may avoid mathematics classes and activities that cultivate the development of quantitative skills and ability because they are low in people-orientation. The lack of development in quantitative skills and ability may further discourage her interest in math-related activities, which in turn impede future learning in these areas. In the long run, the girl may not be equipped with the quantitative skills and ability needed for her to be eligible for or successful in a people-oriented STEM field that she wants to pursue, such as medical science. Therefore, although the level of quantitative ability required in a STEM field was not found to differentially influence men's and women's interests and career choices, interests play a critical role in the early development of quantitative ability. Boys or girls who are disinterested and “turn off” learning in quantitative-related activities are equally unlikely to be successful in pursuing a STEM career later on. As such, (dis)interest *constrains* one's options in educational and occupational pursuits indirectly through affecting ability development.

On the other hand, some researchers have advanced a breadth-based model to explain women's underrepresentation in STEM fields (Valla and Ceci, 2014; also see, Lubinski and Benbow, 2006). The breadth-based model states that females, more likely than males, have interests that promote the development of more symmetrical, competing levels of quantitative and verbal abilities, which in turn afford them with broader career choices. As a result, more females may opt for careers that allow them to express their verbal and people-related skills and abilities, such as law or social sciences, even when they also have the interests and adequate quantitative ability to pursue other STEM fields. This perspective is consistent with empirical findings such as those reported in Woodcock et al. (2013) that people-orientation moderated the relationship between things-orientation and the choice of a STEM major such that students high in things-orientation are less likely to choose a STEM major when their people-orientation is also high. Similarly, Wang et al. (2013) analyzed data from a longitudinal study and reported that mathematically capable twelfth graders who also had high verbal skills were less likely to pursue STEM careers when they were 33 years old than were individuals who had high math skills but moderate verbal skills. Because women were overrepresented in the high math and high verbal skills group, fewer mathematically talented women entered STEM careers compared to their male peers. Therefore, according to the breath-based model, interests do not constrain but rather *broaden* women's career choices through influencing more balanced ability development.

We acknowledge that both processes—*constraining* and *broadening*—may happen in a parallel manner. As discussed earlier in this article and in another paper (Su et al., 2009), individuals engage in both inter-personal and intra-personal comparisons while making educational and career choices. The *constraining* process happens, from the inter-personal perspective, when individuals are selected out or self-select out of STEM fields for not having high quantitative ability compared to other individuals; the *broadening* process happens, from the intra-personal perspective, when individuals evaluate multiple interests and talents within themselves and weigh other options besides STEM careers. Therefore, we urge researchers to examine the indirect effect of interests on the educational and career attainment in STEM fields through learning and ability development in addition to the direct influence of interests on STEM career choices.

### Potential interventions targeting interests and work environments

The current findings have implications for potential interventions to increase women's representation in the STEM workforce. At the individual level, the current findings suggest that interests are critical predictors of occupational membership in STEM fields. Highlighting the societal relevance of STEM knowledge, skills, and careers and their value in improving people's lives may prove to be an effective way for appealing to females' *Social* interests and getting more females to engage in STEM activities (Eccles, 2009; Valla and Ceci, 2014). For example, it has been demonstrated that using a science-technology-society (STS) approach to teaching science in high school improved attitudes toward science, particularly for girls (Bennett et al., 2007). In another experimental study, Harackiewicz et al. (2012) showed that mailing parents brochures about how to help their adolescents in tenth and eleventh grade see the values of mathematics and science to their personal lives increased the adolescents' mathematics and science course-taking by almost one semester. These interventions provide promising ways for educators and parents to increase students' interests and engagement in STEM activities. We note, however, that evidence on the effectiveness of these interventions is still preliminary. More research is needed to quantify the effect sizes of the improvement in students' attitudes, interests, and behaviors, and in particular, the long-term outcomes of the interventions, such as participation in the workforce. We call for more interventions that integrate students' people interests into STEM education and that increase students' perception of task values of STEM activities and careers, as well as more research that uses a longitudinal design to evaluate such interventions. On the other hand, while the literature has consistently shown the influence of social contexts (e.g., parents, schools) on students' interest development, particularly the development of differential interests for boys and girls (e.g., Hartung et al., 2005; Jacobs et al., 2005), little is known about the link between biological factors (e.g., brain structure, hormones) and interest development. To the extent that gender differences in interests are explained by biological factors, the effectiveness of social and educational interventions for increasing girls' interests in STEM fields may be constrained. More research is needed to provide a comprehensive picture of why such large gender differences in interests exist and how they are developed.

Moreover, we note that the timing is important for an intervention that targets individuals' interests, particularly given the relationship between interests and ability development. Few interventions to date have reached individuals before high school, yet the mutual influence between interests and ability development start from a much earlier age. Although little research has examined the career exploration and interest development of preadolescent children, the research that does exist suggests that children do use their interests to guide learning and formulation of career goals before reaching teen-age years (Hartung et al., 2005). For example, a study that surveyed the finalists in the Westinghouse Science Competition and members of the National Academy of Sciences (NAS) showed that the respondents were already certain about their interest in science at an average age of 11 years old, and as early as 4 years old (Feist, 2006). The awareness of interests early on promoted early engagement in research experiences and in turn contributed to the development of scientific talent and lifetime research productivity. Further, children's perceptions of occupations including the traditional sex-type of occupations also start to form during grade school years, which contribute the development of their differential career preferences (Hartung et al., 2005). It was reported that children as young as 4 years of age express occupational preferences along sex-based distinctions (Trice and Rush, 1995). Given this research, we assert that interventions aiming at increasing individuals' interests in STEM fields and reforming individuals' perceptions of STEM careers need to occur at early ages.

Given the importance of interests for individuals' cognitive development and career exploration starting from an early age, it is necessary to assess interests periodically when they begin to form. Just as a standardized achievement test or other types of cognitive assessments that give students, parents, and educators feedback regarding the students' knowledge acquisition and skill development, measuring interests at a regular basis would provide students, parents, and educators with information regarding students' interest development that can be used to guide students' involvement in curricular and non-curricular activities and to facilitate students' career exploration. We propose a national barometer of basic interests to be developed and administered in K-12 education annually. Such an index would be particularly useful for monitoring the development of gender differences in interests and for guiding girls with STEM interests to engage in STEM activities and explore STEM careers.

At the institutional level, work environments in STEM fields can be reconstructed to increase their people-orientation and to better fulfill women's people interests. Although the analyses in the current study used STEM fields as units and focused on the heterogeneity in people-orientation across STEM fields, we note that the work environments within a STEM discipline can vary as well. For example, different universities or different organizations may have different culture, climate, and practices that provide individuals with different experiences. These salient, more proximal environments are likely to have the largest impact on individual behaviors when assessing their fit with individual interests (Holland, 1997). To the extent an academic program or an organization can implement interventions that enhance its people-orientation, such as incorporating mentoring and team-working activities and emphasizing communication (e.g., Seat et al., 2001), women would be more likely to find such work environment congruent with their interests and are more likely to choose and stay in such work environment. More research is needed to examine the effectiveness of such workplace interventions on women's career choice, job satisfaction, and retention in STEM fields.

### Limitations and future research

As we have mentioned earlier, findings from the current study are based on occupational level of analysis and should only be interpreted at the occupational level. An individual level analysis may reveal greater role of quantitative ability for STEM careers, as previous literature suggested. Nonetheless, given findings from the current study, we expect people and things interests at the individual level to strongly influence individuals' career choice and attainment and expect these relationships to account for the effect of gender.

The current study categorized basic interest scales into 13 STEM fields. While we have demonstrated how these 13 STEM fields differ from each other, the basic interest data did not allow us to perform comparisons of STEM occupations at a more refined level. Even within a sub-discipline of STEM, we may still identify occupations that are heterogeneous in terms of their occupational characteristics. For examples, economics and psychology are both nested within social sciences, yet economics is higher in its level of required quantitative ability (4.25 compared to 3.08 for Psychology) and things-orientation (2.33 compared to 1.70) and is substantially lower in its people-orientation (1.67 compared to 5.04). We expect these differences in occupational characteristics to influence the gender differences in interests and the actual gender composition in economics and psychology. Indeed, women constitute a much smaller percentage among the economists compared to psychologists (21.74% compared to 77.42%). We expect the current findings to replicate in future research examining STEM occupations at a finer level.

Lastly, the current findings are correlational and no causal inferences should be made. By conducting a meta-analysis and pooling together many different “slices” at different stages of the developmental process, we partially alleviated the limitations of using cross-sectional data and showed that age did not moderate the size of gender differences in basic interests. However, to truly understand how interests and cognitive ability unfold and interact to influence individuals' career development, more longitudinal studies like Lubinski and Benbow (2006) are needed in the future. To replicate and complement the findings from the current study, experimental studies are needed to establish causal relationships between the things- and people-orientations of a work environment and individuals' interests and career choices.

## Conclusion

To understand the reasons for women's underrepresentation in STEM fields, more attention needs to be paid to interests. In the current study, we showed that women's interests in more people-oriented, and less things-oriented work environments was a key factor that influenced their career choice in STEM fields. Importantly, not only the choices between STEM and non-STEM careers but also the choices within STEM careers reflect individuals' interest patterns. Interventions at the individual level targeting the development of interests and those at the institutional level aiming at creating educational and work environments that better accommodate women's people interests may prove to be fruitful. In addition, findings from the current study highlight the discrepancies in some STEM fields where the number of women did not meet their level of interests, indicating other factors at work. Realizing that the issue of women's underrepresentation is not identical across all STEM fields and the mechanisms contributing to the gender disparities are overlapping yet different is important for designing future investigations and interventions to understand and increase women's representation in STEM using a multivariate approach.

### Conflict of interest statement

The authors declare that the research was conducted in the absence of any commercial or financial relationships that could be construed as a potential conflict of interest.

## Footnotes

^1^We acknowledge that many other factors may underlie women's underrepresentation in STEM fields, including (1) cognition and learning, particularly factors related to mathematical preparation and achievement, (2) developmental environments, such as influences from home, school, and peers, and (3) institutional and organizational biases in the hiring, training, and promotion processes. We focus on interests to offer the evidence for one important, yet less emphasized perspective that explains the differential representation of women across STEM sub-disciplines as well as the overall underrepresentation of women in STEM fields.

^2^Note that there is only one basic interest scale that specifically assessed interest in Computer Science. Therefore, inferential statistics could not be calculated. However, we reported the single effect size as it was from one of the most highly regarded, well-sampled interest inventory—the Strong Interest Inventory (Donnay et al., 2005)—and provided useful information as a reference in the study.

^3^ Syntaxes for calculating these statistics in R are available from the first author upon request.
